# Assessment of Plasma Cystatin C as a Marker of Acute Renal Injury in Patients Undergoing Extracorporeal Shock Wave Lithotripsy for Renal Stone Disease

**DOI:** 10.7759/cureus.67293

**Published:** 2024-08-20

**Authors:** Dineshwar P Singh, Soumya Mondal, Debansu Sarkar

**Affiliations:** 1 Urology, Institute of Post Graduate Medical Education & Research, Kolkata, IND

**Keywords:** creat : creatinine, crp: c-reactive protein, eswl (extracorporeal shockwave lithotripsy), cystatin c, acute kidney injury(aki))

## Abstract

Introduction

This study aimed to assess plasma cystatin C (CysC) as a marker of acute kidney injury (AKI) in patients undergoing extracorporeal shock wave lithotripsy (ESWL). We compared serum levels of CysC, C-reactive protein (CRP), and creatinine before and after ESWL. The study results may have implications for early detection of AKI and prevention of progression to chronic kidney disease.

Methodology

This prospective observational study included 105 adult participants and was conducted from August 2022 to July 2024. ESWL was the only modality of treatment.

Results

Forty-eight (46%) patients developed AKI after ESWL. Patients with AKI had significantly higher post-ESWL mean plasma CysC levels than patients without AKI (121 ± 0.25 vs. 0.94 ± 0.22 mg/dL, respectively; *P* = 0.001). The mean serum CRP levels after ESWL were significantly higher in patients who developed AKI compared with those who did not (4.36 ± 1.63 vs. 2.64 ± 0.95 mg/dL, respectively; *P* = 0.001).

Conclusions

In patients with renal stone disease, serum creatinine, serum CRP, and plasma CysC can be used as markers of acute renal injury after ESWL.

## Introduction

Renal stone disease is a common disorder worldwide with prevalence ranging from 1% to 20% [1]. Extracorporeal shock wave lithotripsy (ESWL) has been a standard treatment for renal and upper ureteric stones of <2 cm since the early 1980s [2]. It is a noninvasive outpatient technique that is safe and easy to do, it can be done with or without anesthesia, and it yields high stone-free rates. Patients tolerate ESWL well, and it has minimal morbidity. However, it has been understood since 1985 that a clinical dose of sound waves causes immediate renal damage that extends from the papilla to the outer cortex, altering renal function in the majority of patients [3]. This focused and predictable harm has two components: a traumatic vascular injury caused by the shock wave’s physical forces and an ischemic/hypoxic reaction due to severely injured renal arteries. Furthermore, an inflammatory reaction known as *lithotripsy nephritis* immediately occurs at the sites of endothelial damage. This oxidative stress renal damage is accompanied by the release of biomolecules that serve as markers of kidney function and inflammation.

Much research has been conducted to assess how ESWL affects renal function [3]. It has recently been proposed that patients with renal failure have increased plasma cystatin C (CysC) levels [4]. CysC is a cysteine protease inhibitor with a molecular weight of 13 kDa that is produced by all nucleated cells. It can be easily tested and serves as an excellent indicator of renal function. It is entirely catabolized in the proximal renal tubule after glomerular filtration and is not returned to circulation.

CysC-based estimates of GFR may outperform those based on creatinine in some patient populations (older adults, children, and patients with transplants, cirrhosis, or malnutrition). CysC has been studied in different populations for the early detection of acute kidney injury (AKI), and serum CysC is more sensitive than serum creatinine (sCr) alone in the early detection of renal failure [5-7]. The CysC-based glomerular filtration rate (GFR) also gives a direct and accurate measurement of GFR independent of age and muscle mass.

CysC appears to be a more dependable marker of early AKI. The GFR in critically ill individuals can change rapidly due to renal hypoperfusion secondary to shock or the use of nephrotoxic agents, but sCr may not change for up to several days [8,9]. This lag could account for the poor diagnostic value of sCr in the early stages of AKI, and thus its limitations for determining the best course of treatment in critically ill patients. Furthermore, sCr is increased in any kidney disease, either acute or chronic.

Although post-ESWL-induced AKI can be accurately measured using real-time GFR, the necessary technology is not widely available everywhere [10]. Therefore, reliable biomarkers are needed for a simple and reliable assessment of kidney tubular damage and AKI following ESWL. In recent studies, several biomarkers have been shown to have the potential to predict AKI following ESWL, but none of them are well-established or without limitations. In the present study, we assessed CysC as a marker of AKI following ESWL and correlated it with risk factors potentially causing AKI in patients post-ESWL. The results of this study may have implications for improving ESWL techniques and patient outcomes.

## Materials and methods

This single-center prospective observational study included 105 patients and was conducted in the Department of Urology of a tertiary care hospital in eastern India from August 2022 to July 2024. The study protocol was approved by the institutional ethics committee (IPGMER/IEC/2022/487).

All patients included in the study had kidney stones and underwent ESWL. Patients fulfilling the selection criteria were briefed about the nature of the study, and their written informed consent was obtained before inclusion. Patients with pre-ESWL sCr of more than 1.4 mg/dL, C-reactive protein (CRP) of more than 10 mg/L, GFR of less than 60 mL/min, pregnancy, uncorrected coagulopathy, untreated urinary tract infection, arterial aneurysm near a stone, obstruction of urinary tract distal to a stone, or calculi >2 cm on presentation were excluded from the study.

Participants’ demographic and clinical data, such as age and sex, presenting symptoms, and clinical findings, were noted. The findings were recorded on the predesigned form. Patients underwent the following routine investigations before ESWL: complete blood count, renal function test, routine urine tests, and microscopy and coagulation profile. The position of the calculi and stone diameters were measured by ultrasonography and plain computed tomography of the kidney, ureter, and bladder. All ESWL procedures were performed using a Dornier Compact Delta II lithotripter. To target the calculi, a combination of fluoroscopy and ultrasonography was used in most patients. Therapy was started at low power and progressively raised during lithotripsy based on the stone breakup, according to the department’s protocol. A shock rate of 60/min was chosen. The total amount of shocks applied in each session was based on attaining adequate calculus fragmentation, with a maximum of 3,000 shocks per sitting. If this number was reached without achieving adequate fragmentation, the procedure was stopped. Plasma CysC, serum CRP, and sCr levels were measured before ESWL, 24 hours post ESWL, and seven days post ESWL. AKI was identified by an increase in sCr of 0.3 mg/dL within 48 hours, an elevation to 1.5 times the baseline level within the first seven days, or a decline in urine output to less than 0.5 mL/kg per hour for at least six hours, following kidney disease: Improving Global Outcome (KDIGO) criteria 2012 [11]. We used these criteria to divide patients post-ESWL into an AKI group and a non-AKI group. Serum levels of CysC, CRP, and creatinine were compared between pre- and post-ESWL time points in both groups. Additionally, the CysC-based estimated glomerular filtration rate (eGFR) was compared with the creatinine-based eGFR.

Sample size calculation

The sample size for the current study was calculated based on a previous study in which the proportion of subjects with AKI was 56.25% as per Raikar et al. and the formula of Lemeshow et al. [12]. Assuming an AKI prevalence of 0.5625 (56.25%), with a precision (δ) of 0.10 (10%) and a type I error (α) of 0.05 (5%), the sample size required was calculated as follows: [1.962 × 0.5625 × (1 − 0.5625)] / 0.102 = 94.54. With rounding, the sample size arrived at for the study was 95. Considering a 10% loss to follow-up, our proposed sample size for the study was 105 to achieve a 95% confidence interval (CI).

Statistical analysis

An unpaired student's t-test was used to analyze the continuous variables. To analyze categorical variables, Fisher’s exact test was used. Nonparametric testing (Wilcoxon-Mann-Whitney U test) was used to analyze the variables that were not normally distributed and to compare groups. Mean and standard deviation (SD) were used to express the data. Significance was defined by *P *< 0.05. GraphPad Prism version 9 statistical calculator software was used to represent data using SPSS, version 15.0 (SPSS Inc., Chicago, IL).

## Results

Out of 105 participants, 66 were men (62.9%) and 39 (37.1%) were women; thus, the male-to-female ratio was 1.7:1. Most of the patients were between 31 and 40 years (32.4%) or between 18 and 30 years (31.4%). The mean (SD) age was 38.13 (12.62) years, with a median age of 38 years (range, 18-84 years). The calculi were found in the renal pelvis in 56 patients (53.33%), the upper calyx in 23 (21.9%), the mid-calyx in 19 (18.1%), and the lower calyx in seven individuals (6.67%). Fifteen patients (14.28%), 63 patients (60%), and 27 patients (25.72%) had calculi ranging in size from 5 to 10, 11 to 15, and 16 to 19 mm, respectively. The largest stone was 2 cm (Table 1).

**Table 1:** Demographic and clinical data of patients.

Demographics	
Total number of patients	105
Male, *n* (%)	66 (62.9)
Female, *n* (%)	39 (37.1)
Age, years (mean ± SD)	38.13 ± 12.62
Calculus size (mm), *n* (%)	
5-10	15 (14.28)
11-15	63 (60)
16-19	27 (25.72)
Location of calculus, *n* (%)	
Renal pelvis	56 (53.33)
Upper calyx	23 (21.9)
Middle calyx	19 (18.1)
Lower calyx	7 (6.67)

AKI occurred in 48 (45.71%) patients based on the 2012 KDIGO criteria for AKI. The patients were then divided into two groups: AKI and non-AKI. A 1.5-fold increase of plasma CysC and serum CRP following ESWL from the baseline value before ESWL was considered to be a significant rise [13-15]. Post-ESWL plasma CysC levels were significantly increased in 61 (58.1%) patients, while 44 (41.9%) of the patients had no significant increase. Post-ESWL serum CRP levels were significantly increased in 64 (61%) patients, while 41 (39%) of the patients had no significant increase. At seven days post-ESWL, CysC remained elevated in seven patients, CRP in 24 patients, and sCr in 18 patients in the AKI group. In the non-AKI group, sCr was increased to significant levels in four patients on the seventh day post-ESWL.

**Figure 1:**
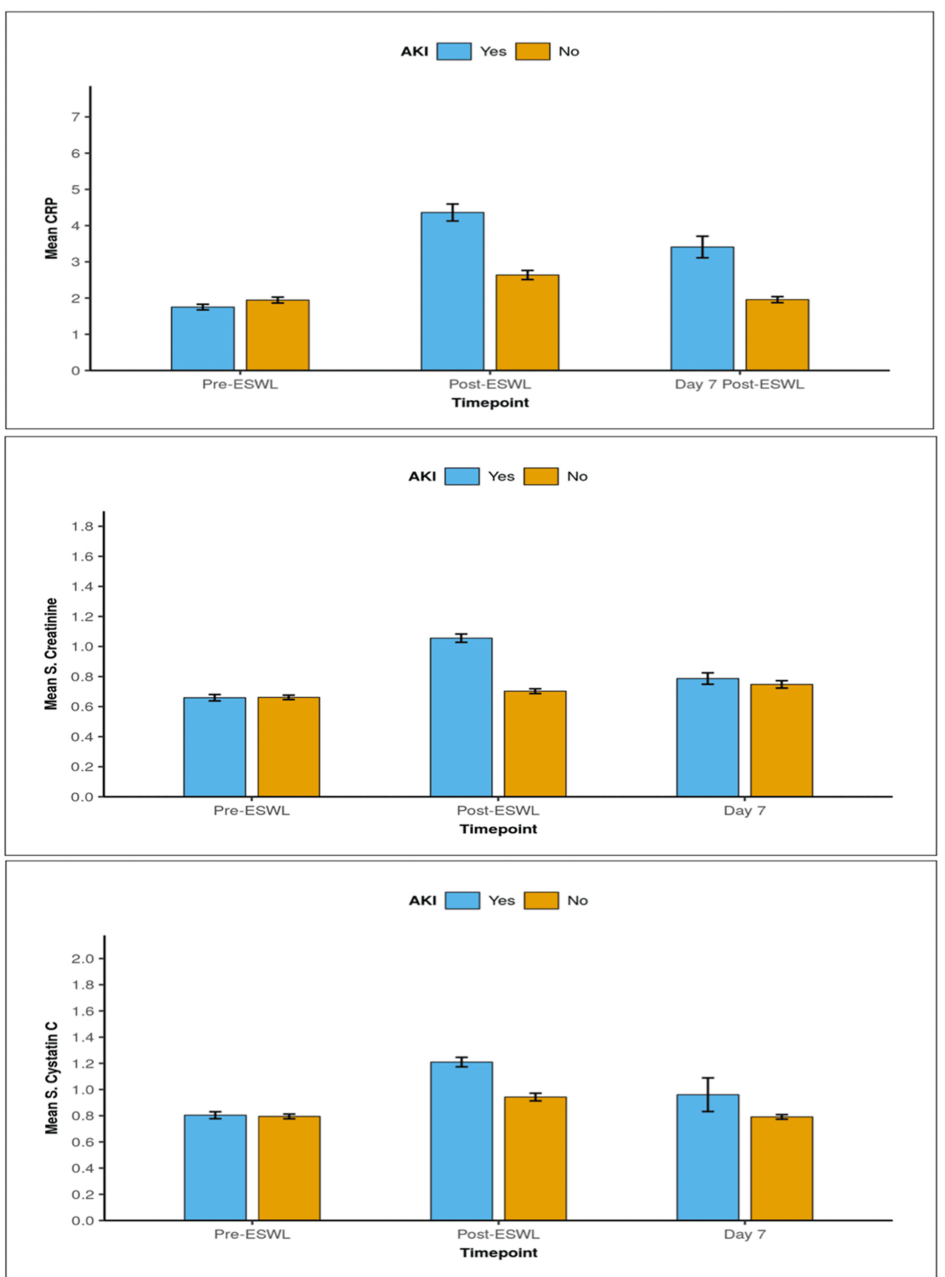
Comparison of the two groups in terms of changes in (a) serum C-reactive protein (CRP), (b) serum creatinine, and (c) serum cystatin C.

Based on the 2012 KDIGO criteria and baseline values before ESWL, the following outcomes were found. In the AKI group, 46 patients (95.8%) showed a significant increase in serum CysC and 47 patients (97.9%) had a significant increase in serum CRP at 24 hours after ESWL. In the non-AKI group, 15 patients (26.3%) showed a significant increase in serum CysC levels, and 17 patients (29.8%) showed a significant increase in serum CRP. The mean plasma CysC levels before ESWL were comparable between the AKI and non-AKI groups (*P* = 0.949); however, individuals in the AKI group had substantially higher post-ESWL mean plasma CysC levels (1.21 ± 0.25 mg/dL) compared with those in the non-AKI group (0.94 ± 0.22 mg/dL; *P* = 0.001). The mean change in plasma CysC levels in AKI patients was significantly higher than in non-AKI patients (*P* = 0.001). The mean plasma CysC levels were again comparable between the two groups at seven days following ESWL (*P* = 0.359; Figure 1).

Patients with AKI had substantially higher post-ESWL sCr levels at 24 hours (1.06 ± 0.19 mg/dL) than those who did not develop AKI (0.70 ± 0.12 mg/dL) (*P *= 0.001). The mean change in sCr levels in AKI patients was substantially higher than in non-AKI patients (*P* = 0.001). At seven days post-ESWL, the mean sCr was comparable between the two groups (*P* = 0.636; Figure 1).

**Figure 2:**
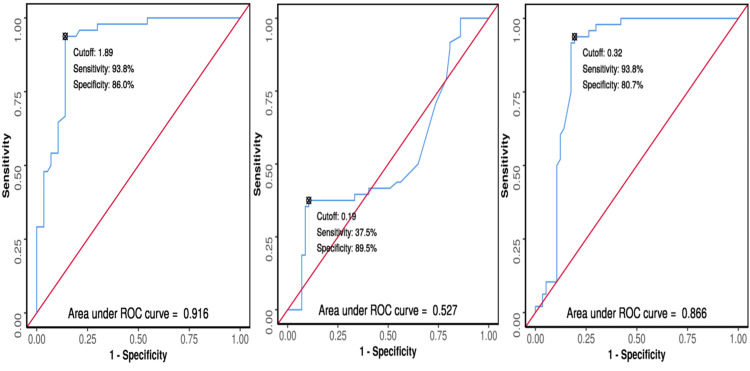
The receiver-operating characteristic (ROC) curve analysis showing diagnostic performance of the changes in (a) serum cystatin C, (b) serum creatinine, and (c) C-reactive protein in predicting acute kidney injury in 105 patients (AKI: Yes vs. AKI: No).

The mean serum CRP levels before ESWL were comparable between the AKI and non-AKI groups (*P* = 0.088). At 24 hours post-ESWL, the mean serum CRP levels were significantly higher in the AKI group (4.36 ± 1.63 mg/dL) than in the non-AKI group (2.64 ± 0.95 mg/dL) (*P* = 0.001). The mean change in serum CRP levels was significantly higher in the AKI group than in the non-AKI group (*P* = 0.001). At seven days post-ESWL, the mean CRP was found to be significantly higher in the AKI group (*P* = 0.002; Figure 2).

The mean CysC-based eGFR before ESWL was comparable between patients who did not develop AKI (108.91 ± 17.02) and those who did (107.12 ± 21.26; *P* = 0.640). The mean CysC-based eGFR post-ESWL was significantly decreased in the AKI group (67.58 ± 16.50) compared with the non-AKI group (93.51 ± 23.67; P = 0.001). The mean change in the CysC-based eGFR was significantly decreased in the AKI group compared with the non-AKI group (*P* = 0.001). The mean sCr-based eGFR before ESWL was comparable between patients who did not develop AKI and those who did (*P* = 0.587). The mean sCr-based eGFR post-ESWL was significantly decreased in the AKI group (84.75 ± 15.96) compared with the non-AKI group (116.65 ± 17.23; *P* = 0.001). The mean change in sCr-based eGFR was significantly decreased in the AKI group compared with the non-AKI group (*P* = 0.001). The mean change in CysC at seven days following ESWL from pre-ESWL levels was similar in both groups, but the mean change of sCr was significantly higher in the non-AKI group and the mean change of serum CRP was significantly higher in the AKI group (Tables 2-3).

**Table 2:** Comparison of differences in serum creatinine between the pre-ESWL time point and the follow-up time points. ^a^Comparison of the two groups in terms of the difference in serum creatinine from pre-ESWL to follow-up time points. AKI, acute kidney injury; ESWL, extracorporeal shock wave lithotripsy; SD, standard deviation

Comparison	Absolute change				*P*-value^a^
	AKI group		Non-AKI group		
	Mean (SD)	*P*-value	Mean (SD)	*P*-value	
24-hour post-ESWL to pre-ESWL	0.40 (0.12)	<0.001	0.04 (0.07)	<0.001	<0.001
7-day post-ESWL to pre-ESWL	0.13 (0.18)	0.276	0.09 (0.18)	<0.001	0.675

**Table 3:** Comparison of differences in C-reactive protein between the pre-ESWL time point and the follow-up time points. ^a^Comparison of the two groups in terms of the difference of CRP from pre-ESWL to follow-up time points. AKI, acute kidney injury; ESWL, extracorporeal shock wave lithotripsy; SD, standard deviation

Comparison	Absolute change				*P*-value^a^
	AKI group		Non-AKI group		
	Mean (SD)	*P*-value	Mean (SD)	*P*-value	
24-hour post-ESWL to pre-ESWL	2.61 (1.34)	<0.001	0.69 (0.74)	<0.001	<0.001
7-day post-ESWL to pre-ESWL	1.66 (1.86)	<0.001	0.01 (0.09)	0.734	<0.001

The area under receiver operating characteristic curve (AUROC) for change in serum CysC predicting AKI versus non-AKI was 0.866 (95% CI 0.788-0.944), thus demonstrating good diagnostic performance. It was statistically significant (*P* ≤ 0.001). At a cutoff of a change in in serum CysC of ≥0.32, AKI was predicted with a sensitivity of 94% and a specificity of 81%. The AUROC for change in sCr predicting AKI versus non-AKI was 0.527 (95% CI 0.411-0.643), thus demonstrating poor diagnostic performance. It was not statistically significant (*P* = 0.638).

At a cutoff of ≥0.19 for the change in sCr, AKI was predicted with a sensitivity of 38% and a specificity of 90%. This cutoff and the diagnostic parameters reported were not reliable, as the test was not statistically significant. The AUROC for change in CRP predicting AKI versus non-AKI was 0.916 (95% CI 0.862-0.97), thus demonstrating excellent diagnostic performance. It was statistically significant (*P* ≤ 0.001). At a cutoff of change in CRP ≥1.889, AKI was predicted with a sensitivity of 94% and a specificity of 86%. Although change in CRP performed slightly better than CysC in terms of specificity, it had a prolonged recovery time and was therefore not optimal for follow-up (Figure 2).

Table 4 provides a summary of the results. In the AKI group, 48 patients had sCr levels above baseline levels at 24 hours post-ESWL, and 18 continued to have significantly increased sCr at seven days post-ESWL. In addition, 46 patients with AKI had raised CysC at 24 hours post-ESWL, with only seven patients still having raised CysC at seven days post-ESWL. Forty-seven patients with AKI had raised CRP at 24 hours post-ESWL and 24 still had raised CRP at seven days post-ESWL. In the non-AKI group, none of the patients had increased sCr at 24 hours post-ESWL, but four patients had raised sCr at seven days post-ESWL. In addition, 15 and 17 of the patients in the non-AKI group had increased CysC and CRP at 24 hours post-ESWL, but levels for both markers returned to baseline by seven days in all these patients.

**Table 4:** Summary of comparison between serum creatinine, cystatin C, and C-reactive protein. ^a^*P*-values indicate significant or nonsignificant changes in AKI markers. Although there were significant increases noted in all the markers in both groups at 24 hours post-ESWL, the mean changes were significantly higher in the AKI group (all having *P*-values < 0.001). AKI, acute kidney injury; ESWL, extracorporeal shock wave lithotripsy; n, number of patients with a significant increase in values of AKI markers

Patient group	Time point post-ESWL	Serum creatinine		Cystatin C		C-reactive protein	
		n	*P*-value	n	*P*-value	n	*P*-value^a^
Both groups	24 hours	48	-	61	-	64	-
	7 days	22	-	7	-	24	-
AKI	24 hours	48	<0.001	46	<0.001	47	<0.001
	7 days	18	0.276	7	0.977	24	<0.001
Non-AKI	24 hours	0	<0.001	15	<0.001	17	<0.001
	7 days	4	<0.001	0	0.471	0	0.734

## Discussion

ESWL is a commonly used procedure for treating patients with upper urinary tract stones who need intervention. It is a noninvasive approach that can be performed as an outpatient procedure [16]. Despite its proven safety and efficacy, complications after ESWL are possible [17,18]. The risk of developing irreversible changes following ESWL is influenced by the number of shock waves, the rate at which shocks are administered, the energy of the shocks, and the number of ESWL treatment sessions. Yet, the relationship between these risk factors and adverse outcomes is not understood [16]. Renal injury markers are needed to enable the prevention of renal injury progression and for early initiation of treatment.

Lagos-Arevalo et al. [14] prospectively studied 160 children with non-cardiac issues admitted to the intensive care unit (ICU). The authors measured CysC and sCr daily, and AKI was staged according to the KDIGO sCr criteria and by similarly applied criteria using CysC (CysC-AKI). They found that 44% of patients developed AKI based on sCr levels, while 32% developed AKI based on CysC levels. Whether AKI was defined by sCr or CysC, neutrophil gelatinase-associated lipocalin was associated with AKI severity. There was no significant correlation between CysC-defined AKI and clinical outcomes; however, early ICU admission CysC levels predicted the development of sCr-defined AKI within 48 hours (area under the curve [AUC] =0.70 [95% CI, 0.53-0.89]) [14]. Similarly, in our study, we used the 2012 KDIGO criteria to define AKI and non-AKI groups. We found that 45.71% of patients developed sCr-defined AKI and 58.1% developed CysC-defined AKI; applying similar principles, we observed that 61% developed CRP-defined AKI.

Zappitelli et al. [15] conducted a three-center prospective cohort study of ICUs in New Haven, CT; Cincinnati, OH; and Montreal, QC, Canada. They concluded that in comparison with the sCr-based definition of AKI, the CysC-based definition was more strongly associated with urine interleukin 18 and kidney injury molecule 1 in children undergoing cardiac surgery. In addition, they suggested defining AKI based on CysC in clinical care and future studies [15]. In our study, we used the same criteria to define AKI and found that post-ESWL CysC-defined AKI occurred in a higher number of patients than sCr-defined AKI, which may have been due to an early rise in serum CysC as also noted in this study and various other studies [19,20].

In a study of 64 patients undergoing ESWL for renal stone disease, Raikar et al. [21] collected measurements for plasma total homocysteine, sCr, and serum high-sensitivity C-reactive protein (hs-CRP) 24 hours before and after the ESWL procedure. Patients were subsequently divided into AKI and non-AKI groups. The authors found that AKI developed in 56.25% of the patients post ESWL. The post-ESWL mean plasma total homocysteine levels were significantly higher in patients with AKI than in those without AKI (21.01 ± 7.67 vs. 16.93 ± 7.44 μmol/L; *P* = 0.036). The post-ESWL mean sCr levels and mean change were also significantly higher in patients with AKI. The mean serum hs-CRP levels after ESWL were similar in patients with or without AKI. However, a substantial increase in serum hs-CRP levels (≥2-fold of baseline) was observed in 72.22% of AKI patients 24 hours after ESWL. The authors concluded that in individuals with renal stone disease, sCr, serum hs-CRP, and plasma total homocysteine can be utilized as markers of acute renal damage after ESWL [21]. In our study, AKI developed in 45.71% of the patients. The 24-hour post-ESWL mean plasma CysC levels were significantly higher in patients with AKI compared with those without AKI (121 ± 0.25 vs. 0.94 ± 0.22 mg/dL, respectively; *P* = 0.001). In contrast to their study, we found that the post-ESWL mean change in serum CRP levels at 24 hours was significantly higher in patients with AKI and in those who did not develop AKI.

Recommendations from the recent Acute Disease Quality Initiative (ADQI) conference [22] advocate distinguishing AKI (first seven days) from acute kidney disease (AKD) (AKI continuing for 7-90 days) and chronic kidney disease (CKD) (beyond 90 days), which may give a framework for measuring recovery in terms of time following the sentinel event. In this respect, 18 out of 48 of our patients failed to recover at the end of seven days post-sentinel event (i.e., ESWL) when sCr was used as the AKI marker. In addition, 24 patients still had raised CRP levels at day 7 post-ESWL; whereas, only seven patients failed to recover when plasma CysC was considered. Furthermore, four patients had Cr-defined AKI at seven days post-ESWL, suggesting a delayed rise in sCr. These four patients also had a significant rise in CysC levels at 24 hours post-ESWL, but these levels fell back to normal at seven days post-ESWL. CRP is an inflammatory marker with a short half-life, and levels usually return to baseline within seven days after the stressor is removed. However, a secondary insult caused by subclinical infection due to bacteria released following stone breakage might cause sustained high levels. Our findings further suggest that there is an early rise and early recovery in plasma CysC post-ESWL, which could identify a crucial therapeutic window and facilitate follow-up (Table 4). Interventions in the form of avoiding Nephro toxic drugs and hydration, which in a patient of AKI prevents further progression to AKD/CKD. Though single kidney status individuals are not included in this study the early rise in plaCysC gives us a rough idea regarding the deteriorating renal function. We can remain cautious and intervene as soon as possible.

Overall, our study’s results point to plasma CysC, CRP, and sCr as potential indicators of acute renal damage. An important limitation of this study was the lack of long‑term follow-up (at least three months) required to be certain that renal function has recovered fully or partially or to determine that it has progressed to CKD. A larger, multi-center investigation with long-term follow-up is needed to confirm our conclusions.

## Conclusions

Although ESWL is considered a safe and effective procedure for treating renal stone disease, it can still lead to AKI. In this study, sCr, plasma CysC, and serum CRP were all significantly increased after AKL caused by ESWL. Therefore, in patients with renal stone disease, serum CRP, sCr, and plasma CysC can be used as markers of AKI after ESWL either individually or in combination.
